# Diabetes Intervention Accentuating Diet and Enhancing Metabolism (DIADEM-I): a randomised controlled trial to examine the impact of an intensive lifestyle intervention consisting of a low-energy diet and physical activity on body weight and metabolism in early type 2 diabetes mellitus: study protocol for a randomized controlled trial

**DOI:** 10.1186/s13063-018-2660-1

**Published:** 2018-05-21

**Authors:** Shahrad Taheri, Odette Chagoury, Hadeel Zaghloul, Sara Elhadad, Salma Hayder Ahmed, Omar Omar, Sherryl Payra, Salma Ahmed, Neda El Khatib, Rasha Abou Amona, Katie El Nahas, Matthew Bolton, Henem Chaar, Noor Suleiman, Amin Jayyousi, Mahmoud Zirie, Ibrahim Janahi, Wahiba Elhag, Abdulla Alnaama, Abduljaleel Zainel, Dahlia Hassan, Tim Cable, Mary Charlson, Martin Wells, Abdulla Al-Hamaq, Samya Al-Abdulla, Abdul Badi Abou-Samra

**Affiliations:** 1 Department of Medicine, Weill Cornell Medicine – Qatar, Doha, Qatar; 2 Joan and Sanford I. Weill Department of Medicine, Weill Cornell Medicine – New York, New York, NY USA; 3 Clinical Research Core, Weill Cornell Medicine – Qatar, Doha, Qatar; 4 Qatar Metabolic Institute (QMI), Department of Medicine, Hamad Medical Corporation, Doha, Qatar; 5 Qatar Diabetes Association, Qatar Foundation, Doha, Qatar; 6 Department of Diabetes and Endocrinology, Hamad Medical Corporation, Doha, Qatar; 7 Primary Health Care Corporation, Doha, Qatar; 8 Aspire Academy, Doha, Qatar; 9 Department of Statistical Science, Cornell University, Ithaca, NY USA; 10 Weill Cornell Medicine – Qatar, Qatar Foundation – Education City, PO 24144, Doha, Qatar

**Keywords:** Type 2 diabetes mellitus, Obesity, Lifestyle, Diet, Physical activity

## Abstract

**Background:**

Type 2 diabetes mellitus (T2DM) and obesity are syndemic and will have a significant impact on affected individuals and healthcare services worldwide. Evidence shows that T2DM remission can be achieved with significant weight loss in those who are younger with early diabetes and requiring fewer medications for glycaemic control. DIADEM-I aims to examine the impact of an intensive lifestyle intervention (ILI) using a low-energy diet (LED) meal replacement approach combined with physical activity in younger individuals with early T2DM.

**Methods:**

The planned study is an ongoing, non-blinded, pragmatic, randomised controlled, parallel-group trial examining the impact of an LED-based ILI on body weight and diabetes remission in younger (18–50 years) T2DM individuals with early diabetes (≤ 3-year duration). The ILI will be compared to usual medical care (UMC). The primary outcome will be weight loss at 12 months. Other key outcomes of interest include diabetes remission, glycaemic control, diabetes complications, cardiovascular health, physical activity, mental health, and quality of life. It is planned for the study to include 138 subjects for assessment of the primary outcome. Safety will be assessed throughout.

**Discussion:**

If DIADEM-I demonstrates a clinically significant effect for younger individuals with early T2DM, it will inform clinical guidelines and services of the future for management of T2DM.

**Trial registration:**

ISRCTN: ISRCTN20754766 (date assigned: 7 June 2017); ClinicalTrials.gov, ID: NCT03225339 Registered on 26 June 2017.

**Electronic supplementary material:**

The online version of this article (10.1186/s13063-018-2660-1) contains supplementary material, which is available to authorized users.

## Background

Obesity and type 2 diabetes mellitus (T2DM) are the greatest challenges to health services worldwide. Obesity is associated with multiple complications including T2DM, hypertension, dyslipidaemia, non-alcoholic fatty liver disease (NAFLD), obstructive sleep apnoea (OSA), cardiovascular disease (CVD), chronic kidney disease (CKD), increased risk for several cancers, poor mental health, and reduced quality of life [1–8]. Overall, obesity is associated with early mortality [2].

Of particular concern is the increasing prevalence of obesity at a younger age. The onset of obesity at a young age results in significant morbidity and mortality [9–12]. In a cohort of young men (*n* = 6502, age 22 years) followed up in Denmark, 48% of those who were obese (Body Mass Index, BMI ≥ 30 kg/m^2^) had developed diabetes, cardiovascular disease, or venous thromboembolism, or had died before the age of 55 years [13]. In a cohort of healthy young men (*n* = 37,674), there was an independent association between elevated BMI at age 17 years and angiography-proven coronary heart disease (β = 1.355, *P* = 0.004) [12].

Diabetes at a younger age is associated with greater risk of devastating macro- and micro-vascular complications [9, 14–16]. In an Australian study [17], the outcomes for 354 T2DM patients with early onset diabetes (aged 15–30 years) were compared to type 1 diabetes (T1DM) patients with a median follow-up of greater than 20 years. Those with T2DM had almost double the mortality compared to the T1DM group (11% vs. 6%). Furthermore, death in the T2DM group occurred after about 10 years’ shorter disease duration than the T1DM group. There were more cardiovascular deaths (50% vs. 30%), and increased neuropathy, in the T2DM group. A study of UK primary care data reported that the adverse cardiovascular risk profiles of the younger-aged (mean age 33.8 years) group were similar to the older group (mean age 66.9 years) [18]. A Swedish population-based study reported that those with T2DM had three times greater risk for severe retinopathy compared to those with T1DM [19].

The current medical approach to T2DM concentrates on symptom control, targeting glycosylated haemoglobin (HbA1c) levels, and prevention of long-term diabetes complications [20]. The combination of intensive glycaemic control and earlier introduction and intensification of insulin treatment has resulted in the development and exacerbation of obesity amongst patients with diabetes [21]. This has created a vicious cycle of obesity and T2DM since greater obesity results in greater requirement for treatments that promote weight gain [22].

Weight loss and lifestyle change are important for managing glycaemia, dyslipidaemia and hypertension after diabetes has developed [23–27]. Weight loss through lifestyle interventions and bariatric surgery has been shown to have a significant impact on T2DM [23, 28–32]. More intensive lifestyle and structured interventions show the greatest benefit for weight loss and glycaemic control [24, 25, 33, 34].

The Look AHEAD study [23–26, 35] examined the impact of an intensive lifestyle intervention (ILI) on cardiovascular outcomes. The study aimed for a weight loss of at least 10% through energy restriction and physical activity [35]. In this study, improvement in glycaemia was associated with degree of weight loss. Furthermore, diabetes remission occurred in 11.5% and 7.3% of the ILI group at year 1 and years 4, respectively, compared to the 2.0% of the control group for both time-points [36]. The ILI group also had a significantly greater percentage of sustained remission, although only 3.5% achieved this at 4 years [36]. The subjects in the Look AHEAD study, however, were older (mean age 58.6 ± 6.8y in the ILI group) and had a median diabetes duration of 5 years (2.0–10 years). A less intensive lifestyle intervention in the UK primary care setting observed modest improvements in glycaemia, but did not observe any diabetes remission [33, 37]. Again, this study was conducted in an older group with mean age of 60.1 (SD 10.2) years for the intervention group. Data from bariatric surgery support the hypothesis that any intervention aiming to have an impact on diabetes remission needs to concentrate on younger patients at an early stage of disease [28, 29, 38–41]. It can be hypothesised that intervention at a younger age and earlier disease stage may result in greater improvement and remission of diabetes.

There is significant evidence that substantial initial weight loss is associated with greater long-term net weight loss [24, 42–45]. A retrospective study of 5965 individuals who undertook a very-low-energy diet (VLED; < 800 kcal/day), the weight lost in the initial weight loss phase was significantly associated with the percentage weight loss maintenance for up to 3 years [46]. In the Look AHEAD study, larger monthly weight loss during the first year, independently predicted weight, HbA1c, HDL-cholesterol, and systolic blood pressure at year 4. Thus, greater initial weight loss is a key factor in weight loss maintenance and distal metabolic outcomes. In the Look AHEAD study, successful lifestyle subjects used meal replacement products and attended more frequently. Also, those who lost most weight and maintained their weight, undertook more physical activity [24, 25, 47].

VLED and low-energy diets (LED) are most effective at achieving substantial initial weight loss. A recent study showed that there was no difference in subsequent weight regain between rapid and gradual weight loss [48]. A meta-analysis of 29 studies from the United States observed that weight loss with VLED resulted in greater weight loss maintenance compared to hypo-energetic balanced diets: 7.1 kg (95%CI: 6.1–8.1 kg) and 2.0 kg (1.5–2.5 kg), respectively [42]. In six studies, those who exercised more, had greater weight loss maintenance [42]. VLEDs have been employed in the last 40 years for rapid weight loss and their use in the management of obesity has been recognised by several guidelines [49]. In a systematic review and meta-analysis, six randomised controlled trials were identified [50], and when VLEDs were compared to LEDs, VLEDs resulted in greater weight loss than LEDs in the short term, but similar long-term weight loss. A recent study has reported that an LED approach is as effective as VLED [51]. LEDs are likely to be better tolerated than VLED by patients because of side effects [52, 53], thus improving diet adherence and reducing patient dropout. Common adverse events related to low-energy diets include dizziness, sensitivity to cold, fatigue, dry skin, halitosis, constipation, diarrhoea, flatulence, gallstones, and hair loss [52, 53].

An early study of insulin-treated type 2 diabetes patients, reported that a VLED approach resulted in significant weight loss and cessation of insulin in some patients [54]. A recent large feasibility study in the UK in patients with extreme obesity also observed that a programme based on a nutritionally complete LED (Cambridge Weight Plan, UK) achieved a significant early weight loss and a substantial weight loss of ≥ 15 kg maintained at 12 months for one third of all patients entering the programme [55]. The subjects in this study were 45.7 ± 10.7 years old with more severe obesity (mean BMI 48.0 ± 7.6 kg/m^2^). No specific physical activity was included. Another recent study showed that 8 weeks of calorie restriction (600 kcal/day) in severely obese patients with type 2 diabetes, normalised pancreatic beta-cell function and improved insulin suppression of liver glucose production [56]. A key finding was the association of beta-cell function improvement with alterations in liver and pancreatic fat. The role of the liver in diabetes is, therefore, important [57, 58].

Physical activity is important for weight loss and its maintenance, and has beneficial cardiovascular effects [59]. The addition of physical activity to energy restriction for weight loss in overweight/obesity was examined in a systematic review [60]. The combination of diet with physical activity resulted in greater weight loss than diet alone with the intervention and after 1 year of follow-up.

A systematic review examined the impact of the combination of diet, aerobic activity, and resistance training for type 2 diabetes prevention. The combination was found to be superior for weight loss and reduction in fasting glucose [61]. A recent systematic review (47 randomised trials with over 8500 subjects) examined different exercise interventions on glycaemic control [62]. Exercise interventions of greater than 150 min/week were found to be most effective. Exercise training consisting of aerobic exercise, resistance training, or both combined was associated with reductions in HbA1c compared to control groups, respectively. Unsupervised exercise combined with dietary intervention was associated with an HbA1c reduction. Another systematic review [63] examined studies that have compared aerobic to resistance exercise in T2DM. Twelve trials (*n* = 626 subjects) were identified with seven having methodological limitations. The majority of the studies were short term (< 6 months). With sensitivity analysis, there was no significant difference in HbA1c reduction between the two exercise approaches.

A recent systematic review examined the impact of combined aerobic and resistance exercise in T2DM, including 14 trials (*n* = 915 subjects). Aerobic exercise was more effective than resistance training in HbA1c reduction. Combined treatment, however, was superior to aerobic exercise [64]. The same group carried out a systematic review of long-term lifestyle interventions, reporting that the combination of diet and physical activity was superior to all other modalities resulting in an improved lipid profile and reduction in blood pressure, conferring additional cardiovascular benefit [65].

In summary, the combination of physical activity with diet results in greater weight loss and metabolic improvements. Aerobic exercise has greater and more predictable effects on HbA1c. The combination of aerobic exercise with resistance training may be superior the aerobic exercise alone, but more evidence is required. Based on current evidence, the American College of Sports Medicine and American Diabetes Association (ADA) joint position stand [66, 67] and other guidelines [68] recommend a combination of aerobic and resistance exercise.

### DIADEM-I Study rationale

Emerging evidence suggests that it is time to rethink the medical approach to patients with T2DM. Patients with obesity and T2DM who have achieved significant weight loss through either an ILI or bariatric surgery can have remission of diabetes, and improved quality of life. ILI in the Look AHEAD study resulted in a modest diabetes remission, but the study population had an average age of about 60 years, and longer duration of diabetes. There is extensive evidence that the use of LEDs, and particularly those with low carbohydrate content, can safely result in significant weight loss. There is also evidence to support a role for physical activity in improving diabetes. It is now time to put this evidence into practice through a randomised controlled trial to investigate whether combining energy restriction with physical activity in younger patients at an early stage of T2DM can improve weight loss and diabetes outcome measures compared to usual medical care, and to examine whether diabetes remission can be achieved. Tackling obesity and diabetes at the earliest stage (younger patients with short diabetes duration) is critical to reducing disease burden with the potential important benefit of reversing diabetes, and preventing its long-term sequelae. The DIADEM-I study aims to implement an ILI incorporating an LED approach in younger T2DM patients with early diabetes.

### Hypothesis

The key hypotheses of DIADEM-I are that an ILI combining an individualised LED approach with physical activity in subjects with early T2DM, compared to the usual medical care (UMC) group, will result in:

1. Greater weight loss
2. Improvement in glycaemic control
3. Potential remission of diabetes
4. Improved quality of life

### Primary objective

The primary objective of DIADEM-I is to examine the effectiveness of an individualised ILI combining an LED approach and gradual introduction of food with physical activity in younger subjects with early T2DM. The overall aim is to test an intervention that will be successful in weight loss and potential diabetes remission. Remission of diabetes will be defined as:

1. HbA1c outside the diabetes range (< 6.5%)
2. No pharmacologic therapy for diabetes

The ILI will be compared to UMC. The study will examine the primary outcome of weight loss.

### Secondary objectives

The study aims to assess the effects of an LED and physical activity ILI on other important diabetes outcomes. Secondary outcomes of interest will include body composition and energy expenditure (measured by bioimpedance and indirect calorimetry) and weight loss maintenance, markers of diabetes improvement (HbA1c; measured at a centralised laboratory using turbidimetric inhibition immunoassay for haemolyzed whole blood in ROCHE COBAS 6000c Module), insulin sensitivity/resistance (homeostatic model assessment, HOMA), glucose homeostasis (continuous glucose monitoring system, CGMS) [69], cardiovascular health and risk (blood pressure, arterial stiffness, and endothelial function [70]), impact on diabetes complications (nephropathy, neuropathy, and retinopathy) and liver function tests; steatosis (Fibroscan)), changes in physical activity (measured by questionnaire (International Physical Activity Questionnaire IPAQ and Sedentariness Questionnaire, SIT-Q-7-D [71])] and accelerometry (GT3X) [72]), quality of life (Impact of Weight on Quality of Life (IWQoL-Lite) [73] and EQ-5D questionnaires), and mental health (Hospital Anxiety and Depression Scale (HADS) [4, 74]). Furthermore, the study will investigate any side effects and adverse outcomes of the intervention. Biological samples will be collected for metabolomics, proteomics, non-coding RNA, and microbiome. Fasted venous blood samples will be collected by trained phlebotomists.

### Outcome measures

Table 1 lists key outcome measures and instruments (validated for the study population) used for their measurement.

**Table 1:** Key measures and instruments

Key Measures	Instruments	Notes
Body weight and composition	TANITA scales BC420MA	Body weight, and bioimpedance for body composition.
Height	Stadiometer SECA 123	
HbA1c	Turbidimetric inhibition immunoassay for haemolysed whole blood in ROCHE COBAS 6000c Module	Measured at a central laboratory.
Continuous Glucose Monitoring	iPro2 Professional CGM system (Medtronic MiniMed)	Continuous glucose monitoring system (CGMS) will be used for 7 consecutive days.
Quality of Life	1. EQ-5D-5 L 2. IWQOL-LITE	1. EQ-5D-5 L comprises of five dimensions: mobility, self-care, usual activities, pain/discomfort and anxiety/depression. Each dimension has five levels: no problems, slight problems, moderate problems, severe problems and extreme problems. 2. Impact of Weight on Quality Of Life-Lite (IWQOL-Lite) is a validated 31-item, self-report measure of obesity-specific quality of life. In addition to a total score, there are scores on five domains: physical function; self-esteem; sexual life; public distress; work.
Mental Health	HADS	The Hospital Anxiety and Depression Scale (HADS) comprises 14 items, 7 for anxiety, and 7 for depression. The scale yields individual anxiety and depression scores as well as an overall HADS score.
Physical Activity	1. IPAQ 2. SIT-Q-7D 3. ActiGraph GT3X	1. The International Physical Activity Questionnaire (IPAQ) comprises of seven questions recalling the previous week’s physical activity falling into four different physical activity domains: leisure-time activities, domestic and gardening activities, work-related activities, and transportation. 2. The Sedentary Behaviour Questionnaire (SIT-Q-7d) is a self-report recall measure of sedentariness over the last seven days. It assesses total daily sedentary time as an aggregate of sitting/lying down in five domains (meals, transportation, occupation, non-occupational screen time, and other sedentary time). 3. The ActiGraph GT3X is a wrist-worn activity monitor that uses triaxial accelerometry to collect physical activity and sleep data. Accerometry will be carried out over 7 consecutive days.
Energy Expenditure	Indirect calorimetry (Cosmed Fitmate GS)	Portable desktop indirect calorimeter for measurement of Resting Metabolic Rate (RMR)
Liver transient elastography	EchoSens Fibroscan 502 Touch	Non-invasive elastography device which gives 50 Hz shear wave speed measurements and estimates of liver stiffness as well as 3.5 MHz ultrasound coefficient of attenuation.

## Methods

DIADEM-I is an ongoing, non-blinded, randomised controlled, parallel-group trial examining the impact of an ILI using LED and physical activity for younger subjects with early T2DM.

**Fig. 1:**
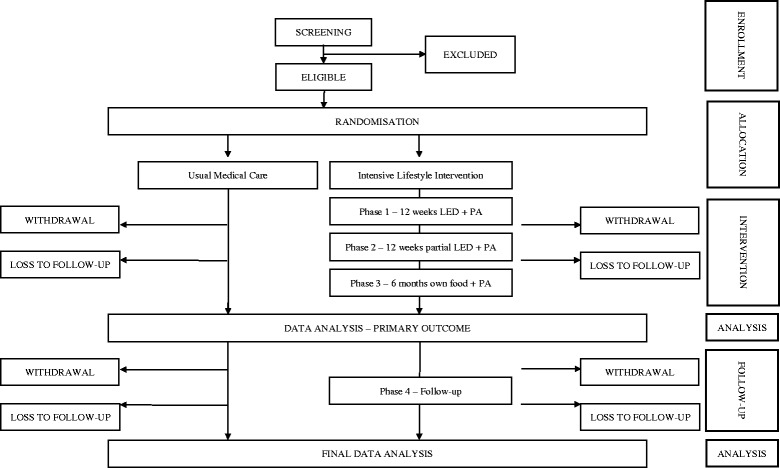
CONSORT flow chart for DIADEM-I Study. LED = Low Energy Diet; PA = Physical Activity

**Fig. 2:**
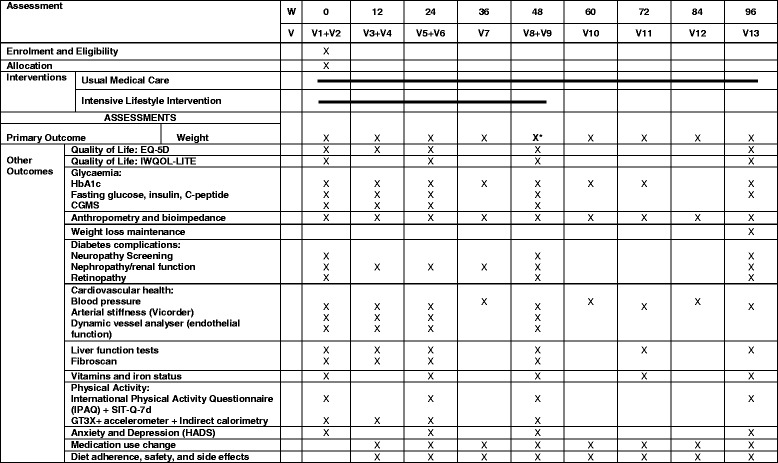
Schedule of enrolment, intervention, study visits and assessments for both study groups

The trial is registered with ISRCTN ISRCTN20754766 (date assigned: 7 June 2017), and ClinicalTrials.gov identifier (NCT03225339 (registered on 26 June 2017).). The description of the protocol is based on the latest version of the study protocol (DIADEM-I STUDY PROTOCOL v3–7 December 2016). The reported protocol follows the SPIRIT (Standard Protocol Items: Recommendations for Interventional Trials) recommendations (http://www.spirit-statement.org/). The SPIRIT checklist is provided in the Additional file 1. Figure 1 shows the CONSORT flow chart. Figure 2 shows the schedule of enrolment, intervention, study visits and assessments for the study groups.

### Sponsor and funding

The study is sponsored by Weill Cornell Medicine – Qatar and funded by the Qatar National Research Fund (QNRF) through the National Priorities Research Program (NPRP) grant NPRP 8–912–3-192. Support is also provided by the Clinical Research Core at Weill Cornell Medicine in Qatar, supported by the Biomedical Research Program (BMRP; funded by Qatar Foundation). There is no input from the funding or sponsor organisations into the design, conduct, analysis, or reporting of the study.

### Ethical approvals and monitoring

Ethical approval for the study has been obtained from the Weill Cornell Medicine – Qatar Institutional Review Board (IRB), Hamad Medical Corporation – Qatar IRB, Primary Health Care Corporation (PHCC) – Qatar IRB, and the Ministry of Public Health, Doha, Qatar. The study is supported by the Weill Cornell Medicine – Qatar institutional Data and Safety Monitoring Board (DSMB). Study monitoring will be conducted at 3-monthly intervals by the Clinical Research Core, Weill Cornell Medicine – Qatar following a standardised protocol.

### Study design

DIADEM-I is an open-label, pragmatic, parallel-group RCT, employing the following interventions (Fig. 2):

1. Intensive Lifestyle Intervention (ILI) combining a low-energy diet (LED) approach with physical activity. Behavioural support for the lifestyle intervention will also be provided
2. Usual medical care (UMC) aiming at diabetes education and support, and medical management of glycaemia, hypertension, and dyslipidaemia. This will be based on current clinical practice aiming to reduce diabetes symptoms and complications, and general recommendations on diet and physical activity.

### Description of Intensive Lifestyle Intervention (ILI)

The primary objective is for subjects to achieve a significant reduction in body weight at 12 weeks and continue progress at 1 year following a diet and physical activity ILI, and maintain this during the follow-up period. Diabetes remission will be assessed throughout the study.

#### Diet

Subjects are supported by a trained dietitian. The study will use a commercially available meal replacement plan – the Cambridge Weight Plan [51, 55, 75–78]. The ILI will use 800 kcal as a benchmark. For the first 12 weeks, subjects will be asked to consume mainly meal replacement products, supplemented by low-fat milk, to make total energy of approximately 800 kcal. Subjects will receive the meal replacement products, free of charge. If there is a need for additional snacks because of hunger, subjects will be recommended to eat raw vegetables and salad. For constipation, a common side effect, subjects will be recommended a fibre supplement (psyllium/inulin), if required. They will be advised to drink ≥2 l of water daily. From month 4 to month 6 inclusive, subjects will follow a partial meal replacement plan and will be introduced to normal solid foods providing daily energy-based on body weight as recommended in the Look AHEAD study. During the food introduction phase, focus will initially be on introducing protein-rich foods [79], skimmed milk, vegetables and salad. Then gradually, subjects will establish a three-meal-per-day eating pattern with support from the dietitian to help them identify appropriate acceptable foods (and portion sizes) to facilitate reintroduction of breakfast, lunch, and evening meal, and snacks. Recipes and meal plans will be provided and the emphasis will be on low-glycaemic-index foods. In the next 6 months, subjects will continue with lifestyle change and increasing physical activity. There will be a further 12-month follow-up period with subjects implementing lifestyle change. To aid self-monitoring, subjects will be provided with a personal activity device.

#### Physical activity

A fully trained exercise trainer will advise subjects in the activity arm to improve their fitness levels by engaging in unsupervised physical activity of at least 150 min per week. Previous studies have shown that at least 150 min of activity per week is effective for an impact on diabetes [66–68]. The planned ILI will follow the American College of Sports Medicine and American Diabetes Association position stand regarding physical activity [67]:

1. Minimum of 150 min/week of exercise of at least moderate intensity
2. Aerobic exercise 3 days/week with no more than two consecutive days between bouts
3. Resistance exercise at least twice weekly on non-consecutive days
4. Gradual rate of progression

The initial focus of activity is to reduce sedentariness, and encouragement of walking. For this reason, those unable to walk due to arthritis will be excluded. Walking activity will aim to achieve to at least 10,000 steps/day. As subjects progress, other aerobic activities and resistance training will be introduced. The objective is to introduce activities that are enjoyable to subjects, thus ensuring sustainability. Higher physical activity levels will be encouraged during the study for optimal weight loss, weight loss maintenance, and diabetes control. Increase in physical activity will be individualized based on progress. Potential benefits of variation in exercise activities will be pointed out [67].

#### Medications

It is aimed that diabetes medications will be discontinued with the adoption of LED. The process and rate of reduction of any medication will be judged clinically and will depend on symptoms, glycaemic control at baseline, and the subject’s motivation to adopt the LED. Blood pressure medications will be discontinued based on clinical assessments. For the UMC group, any medication intensification will aim to choose diabetes medications that either promote weight loss or are weight neutral.

#### Behavioural support

The behavioural aspects to support the dietary and activity intervention will be delivered by fully trained dietitians as well as physical activity trainers. These will be embedded within individual dietetic and activity appointments. Table 2 shows examples of the techniques employed in clinical weight management. The dietitians and physical activity trainers will follow a strict manual of procedures to cover all aspects of behaviour modification support including individualisation to address specific subject challenges and requirements. They will use specific presentations to ensure standardisation of delivery.

**Table 2:** Behavioural support and self-management strategies [87] employed to support the lifestyle intervention in DIADEM-I

Behavioural support and self-management strategies	Examples of behavioural techniques
Self monitoring	• Identifying the relationship between mood and food selection and intake this will be accomplished using a food and mood diary • Using scales to gauge hunger and satiety • Regular weight measurement • Recording of food selection and intake
Goal setting	• SMART (Specific, Measureable, Achievable, Relevant and Time-Bound) goals will be used throughout • Individualised goals will be set by participants regarding diet and physical activity
Stimulus control	• Identification of external and internal triggers to unhealthy eating habits • Use of distraction techniques to help subjects avoid emotional eating
Cognitive restructuring	• Reviewing the impact of negative thoughts and beliefs and their impact on behaviour change. • Replacing negative thoughts and beliefs with positive thoughts and beliefs
Eating behaviour	• Education about healthy eating behaviours • Choosing low energy alternatives to high energy foods • Portion control • Timing and duration of eating • Dealing with snacking
Planning	• Planning meals and physical activity • Planning for challenging situations e.g. eating with family, eating at restaurants, and eating and activity while traveling
Maintenance	• Maintaining successful behaviours • Dealing with lapses and avoiding relapse • Avoiding previous behaviours that resulted in weight regain
Reward & support	• Non-food related rewards • Support from family and friends
Problem solving	• Problem solving skills • Dealing with high risk situations that impact on eating

#### Intervention duration and follow-up

The intervention will be for 12 months for the primary outcome measure with 12 months further follow-up.

The 2 study arms will have the following schedule:

i. Intensive Lifestyle Intervention Group:

- Intervention
  - PHASE 1: 12 weeks LED + physical activity;
  - PHASE 2: 12 weeks partial LED + physical activity;
  - PHASE 3: 6 months own food, physical activity, and lifestyle change.
- Follow-up
  d. PHASE 4: 12 months follow-up;
  e. PHASE 5: Post-study follow-up via electronic medical records (including body weight, medications, and biochemical measures).

ii. Usual Medical Care Group:

- There will be 12 months usual care (optimising individualised glycaemic, blood pressure, and lipid control) with standard diet and activity advice, and a further 12 months follow-up. Contact frequency will be based on the current recommended usual care 3-monthly clinic attendance. To maintain participation in the study, the subjects in this group will receive regular monthly contact during the intervention from the research team via telephone and electronically in between formal visits.

#### Schedule of visits (ILI)

Table 3 shows the schedule of intervention visits for the ILI group.

**Table 3:** Study phases and visit schedules for the intensive lifestyle intervention

Phase	Timeline	Individual Visit Schedule
1. Low Energy Diet + Physical Activity	Weeks 1–12	Visit every 2 weeks Dietitian (30 min/visit) Exercise trainer (30 min/visit)
		Physician review at baseline and on completion of Phase 1
2. Partial Low Energy Diet + Physical Activity	Weeks 13–24	Visit every 2 weeks Dietitian (30 min/visit) Exercise trainer (30 min/visit)
		Physician review on completion of Phase 2
3. Lifestyle change	Weeks 25–48	Visit every 4 weeks Dietitian (30 min/visit) Exercise trainer (30 min/visit)
		Physician review at 3-month and on completion of Phase 3
4. Follow-up	Weeks 49–96	3 monthly visits for follow-up Dietitian (30 min/visit) Exercise trainer (30 min/visit)
		Physician review at 3-month intervals and completion of Phase 4
5. Post Study	Minimum 2 years post-study	Review by weight management service Collection of data via electronic medical records

### Recruitment and Setting

The aim of the study is to assess the effectiveness of the ILI within the primary care setting. Research visits will occur in primary care, although usual care will be conducted either in the primary or secondary care setting depending on the participant’s diabetes care provider. Individuals will be identified, screened for eligibility and recruited from primary and secondary care in Qatar. Participants will be identified by their healthcare team in the outpatient department, and if they are interested in participating in the study, they will be scheduled for a screening visit. The referring clinician will ensure that all clinically routine blood and urine tests have been conducted to allow assessment of participant eligibility and participation. Informed consent is taken by a trained member of the research team independent of the referring physician.

### Eligibility criteria

The eligibility criteria are designed to include subjects appropriate for the study protocol. All relevant medical and non-medical conditions will be taken into consideration by the investigator team on whether the protocol is appropriate for an individual subject.

#### Inclusion criteria

The following inclusion criteria apply:

1. Established physician diagnosed type 2 diabetes mellitus based on American Diabetes Association (ADA) criteria;
2. Diabetes of ≤3-year duration;
3. BMI > 27.0 kg/m^2^ (based on WHO cut-points for ethnicity [80]);
4. Men and women;
5. Age 18–50 years;
6. Originating from the Middle East and North Africa region and resident in Qatar (to ensure diet homogeneity);
7. Able to commit to the study duration;
8. Able to give informed consent and willing to participate in the study.

#### Exclusion criteria

Subjects will not be eligible for enrolment in the study if they fulfil any of the following criteria:

1. Type 1 diabetes mellitus based on clinical history;
2. Cardiovascular event in the previous 6 months;
3. Chronic kidney disease stage 3b or greater (estimated glomerular filtration rate eGFR < 30 mL/min/1.73 m^2^);
4. Currently pregnant, lactating, or planning pregnancy within the study period;
5. Any condition precipitating fluid overload such as heart failure (NYHA class > I) and liver cirrhosis;
6. Significant previously diagnosed psychiatric disorder (e.g. schizophrenia, post-traumatic stress disorder, obsessive-compulsive disorder);
7. Uncontrolled depression (based on hospital anxiety and depression scale);
8. Uncontrolled epilepsy;
9. Known lactose intolerance;
10. Severe arthritis preventing walking;
11. Active gout;
12. Active gallstone disease or known asymptomatic gallstones.

These exclusion criteria also include conditions that may be affected by use and composition of a low energy meal replacement diet e.g. lactose intolerance. Cardiovascular events are ischaemia-related events (e.g. myocardial infarction, angina, transient ischaemic attack, and stroke).

### Subject adherence, withdrawal, safety measures and adverse events

Adherence will be measured at each visit. Subjects will be asked about their dietary intake and physical activity. Subjects in the ILI will be asked to return any empty packets or unused meal replacement products to examine usage. Subjects who are found to be non-adherent or withdraw in the first 6 weeks of the study will be replaced.

The subject has the right to withdraw their consent anytime without giving any reason. The subject’s reason for withdrawal (if reported) will be recorded. Subjects will be notified that if they choose to withdraw from the study, the investigative team will not proceed with further assessments and data collection but their previously collected data will be used for research purposes. Withdrawal from the study will be recorded in the electronic medical record.

Also, the study will withdraw the subject if:

- The subject is unable to adhere to the study protocol and study requirements;
- There is a significant protocol deviation;
- The subject becomes pregnant during the study;
- Any new illness that affects their inclusion (exclusion criteria above);
- Continuing in the study is deemed harmful to the subject’s health;
- The subject is lost to follow up;
- The trial cancels.

All female subjects will be asked to report if they miss any menstrual cycle. A urine pregnancy test will be conducted and subjects withdrawn if the test is positive.

Any harm that may result in a subject’s withdrawal, or is present at the end of the study, will be followed up until a satisfactory resolution occurs. Safety measures include home capillary blood glucose monitoring (for hyper- and hypoglycaemia), blood pressure measures, electrocardiogram at baseline (to identify any covert cardiac disease that may affect physical activity), and laboratory measures to assess any blood, liver, renal, or nutritional abnormalities. Specific advice will be given regarding home blood glucose monitoring for hypo- and hyper- glycaemia, as well as other foreseeable changes associated with weight loss or dietary change and increased activity. Medications that may increase risk of adverse effects will be discontinued.

All adverse events (AE) will be collected and reported using specific forms. At every visit, the subject will be asked about any experiences by asking: “since your last visit, have you experienced any problems?” All AEs will be recorded and evaluated by the Investigators.

### Intervention fidelity

Intervention fidelity will be ensured through regular training of the research team who will use a standardised program to ensure that all subjects in the ILI receive uniform education, training and intervention. The delivery of the intervention will be assessed through regular observation.

### Case report forms and data entry

Subjects will be asked for permission to obtain their clinical information from their electronic medical records. Paper case report forms (CRFs) will be completed for each visit. Each CRF will be validated by a research investigator. The CRF data will be translated into electronic data. All data will be double entered into a secure electronic database in preparation for data analysis.

### Sample size calculations

The sample size is based on the number of subjects needed to provide a power equal to 0.8 to test the primary hypothesis at the 0.05 significance level. The primary outcome for the proposed study is weight loss at 12 months. We anticipate that the weight loss will be 7% higher in the lifestyle group compared to the usual care group at 12 months. The Look AHEAD study yields a conservative estimate of the standard deviation of percentage of weight loss across various populations to be 9%. Using ANCOVA method to calculate the sample size and with 30% inflation for dropouts, 69 subjects per group are needed for the primary outcome. Regarding glycaemic control, Unick et al. found a − 0.643 (± 0.99) % change [81]; however, a change of 0.5% is considered to be clinically significant and comparable to changes achieved with most diabetes medications. The 69 subjects per group is also sufficient to detect a 0.5% change in HbA1c (assuming SD = 1%; the same power, significant level, and dropout rate). The study will therefore aim to recruit a total of 138 subjects (69 per group) including a 30% dropout.

### Subject replacement

Any subject who drops out or is withdrawn in the first 6 weeks of study participation will be replaced. We estimate about 10% of recruited participants will be in this category.

### Randomisation and allocation concealment

Consented eligible subject will be randomly allocated to either receive intensive lifestyle intervention or usual medical care. Allocation will be made in a 1:1 ratio via a web-based system that uses a computer-generated randomisation list with variable block sizes (2, 4, and 8). The allocations are computer generated in Stata (version 13.1) by the trial statistician and programmed into an online randomisation service (https://www.sealedenvelope.com/) to which the trial coordinating team and investigators have no access.

When the subject has been recruited to the study, the subject will be allocated a study number, given sequentially; starting at one and the subject’s baseline data will be collected. Once this has occurred, only the person recruiting the subject will be informed of the group that has been randomly assigned to that study number. If a subject drops out of the study, the assigned study number will not be reused.

### Data analysis

Once all data are entered, multi-item scales will be computed and the univariate distributions of all outcome measures and potential covariates and confounders will be examined. If out-of-range values are found, we will attempt to determine the correct value or, failing this, the value will be treated as missing. Data will be expressed as frequencies and percentages for categorical variables, mean and (±) standard deviation for continuous variables or as median accompanied by interquartile range (IQR) for skewed continuous variables, as appropriate. Depending on the distribution of the data, independent student t-tests or Mann-Whitney U tests will be used to compare outcomes between the groups. Categorical baseline characteristics were compared using Chi squared tests. It is anticipated that some measures will exhibit substantial positive skew, and for these, we will determine if a logarithmic or power (e.g. Box-Cox) transformation is able to reduce the deviations from normality for those analyses involving the continuous version of the variable.

The main objective of the proposed study is to examine the effectiveness of an ILI. The ILI will be compared to usual care. The study will examine the primary outcome of weight loss, and also focus on improvement in glycaemic control. This will be assessed using an ANCOVA model. The first response variable will be weight at one year. The covariates will be weight at baseline and a binary variable indicating whether the subject was in the intervention group or the usual group. The regression coefficient of the group term represents the effect size of the intervention. In addition to this unadjusted analysis, an adjusted analysis will also be performed by adding body fat, fat free mass, HbA1c, and medication as covariates in the model. Similar unadjusted and adjusted ANCOVA models to assess glycaemic control using HbA1c will be fit. The primary analyses will be done using the intention-to-treat (ITT) method. Secondary outcomes (body composition and weight loss maintenance, insulin sensitivity/resistance, cardiovascular health and risk, impact on diabetes complications and liver function, changes in physical activity, mood, and quality of life) will be analysed in the same way, using unadjusted and adjusted analyses, under the ITT principle. Continuous measures will be analysed using an ANCOVA model and categorical outcomes using the appropriate generalised linear model, with the baseline measure of the outcome as a covariate. All outcome measures will also be analysed with per protocol.

In case of missing values, data imputation as well as comparison between imputed and non-imputed scores will be undertaken. A macro will be written that, for each measure, 1) identifies each pattern of missing data that occurs for the items used to construct the measures, 2) performs a separate regression analysis for each pattern predicting the measure value, for those with complete data, from the non-missing items for that pattern, and 3) uses the regression equation to replace the missing measure value with the regression-based predicted score for people having that pattern of missing data. Missing data are only replaced with predicted scores when the regression analysis indicates that the predicted score is a good estimate (i.e., when R-squared > 75% for the regression equation; almost always the case except for those who are missing more than 50% of items). Subgroup analysis will be performed gender and baseline BMI (< 30 and > = 30 kg/m^2^).

## Discussion

There is a close relationship between T2DM and excess adiposity. Current evidence suggests that weight loss through medical interventions or bariatric surgery can result in diabetes remission. However, a medical approach to diabetes remission has rarely been tested rigorously. Evidence suggests that T2DM patients who are younger with shorter duration of diabetes are most likely to achieve diabetes remission.

The novelty of DIADEM-I is in addressing diabetes in younger individuals at an early stage of diabetes aiming to improve diabetes significantly or potentially putting it into remission. Most studies have examined interventions in older patients at a more advanced disease stage. Younger patients are more likely to adopt physical activity [82]. Another novelty of the study is the use of an LED approach to achieve significant initial weight loss, to gradually introduce food intake, and to develop physical activity alongside the diet.

There is increasing evidence that significant improvements and T2DM remission can occur through medical interventions using a low energy diet approach. Recently, the DiRECT open-label cluster randomised primary care trial reported outcomes of an LED intervention for T2DM remission [83]. The study observed that at 12 months, 24% of the LED intervention group achieved 15 kg weight loss with nearly a half (56%) achieving diabetes remission. There are key differences between DIADEM-I study and DiRECT. DiRECT adopted a cluster randomised approach, while DIADEM-I employs an individual randomisation approach. Contamination is avoided in DIADEM-I through independent appointment days for the trial group. DIADEM-I also specifically targets those who are younger with shorter duration of diabetes, although the duration of diabetes in the DiRECT study was about 3 years. DIADEM-I emphasises physical activity with input from exercise trainers. Another ongoing study is examining the impact of an LED approach on type 2 diabetes patients who are on insulin treatment (ISRCTN2133588). This study could extend the range of patients who may benefit from an LED approach.

DIADEM-I is a major collaboration amongst multiple institutions in Qatar. A key strength of conducting DIADEM-I in Qatar is the increasing availability of Wellness Centres in primary care providing access to exercise facilities. The smaller geographical area allows easy access to facilities. The high prevalence of obesity and T2DM in families could result in downstream lifestyle improvements in family members not participating in the study. There are, however, specific challenges in conducting a randomised controlled trial in Qatar. The population has limited exposure to randomised clinical trials [84, 85]. DIADEM-I will report on recruitment and retention rates that will inform future clinical research studies in Qatar. Another consideration is that the population in Qatar tends to be transient with many expatriates. This may affect the duration of follow-up within the study to examine long-term impacts. Nevertheless, Qatar has one of the highest prevalence of diabetes in the world affecting a younger population, and it is essential to implement interventions based on local evidence. Furthermore, there is great recourse to bariatric surgery in Qatar and worldwide when medical interventions may be just as effective.

The findings from the DIADEM-I study will be disseminated through peer-reviewed publication, following ICMJE recommendations (http://www.icmje.org/), and results presented at international meetings to healthcare professionals. Further dissemination will be through the internet and social media [86]. It is expected that findings from DIADEM-I will be incorporated into patient care guidelines and be translated into future services for patients with T2DM.

## Trial status

The trial is currently underway. It is planned for the study to complete by May 10, 2019. Any protocol amendments will be updated in ISRCTN and clinicaltrials.gov.

## Additional file

Additional file 1: SPIRIT 2013 Checklist: Recommended items to address in a clinical trial protocol and related documents*. (DOC 124 kb)

## Abbreviations

ADA: American Diabetes Association
AE: Adverse event
ANCOVA: Analysis of covariance
BMI: Body Mass Index
BMR: Basal metabolic rate
CGMS: Continuous glucose monitoring system
CKD: Chronic kidney disease
CRF: Case report form
CVD: Cardiovascular disease
DSMB: Data and Safety Monitoring Board
ECG: Electrocardiogram
eGFR: Estimated glomerular filtration rate
HADS: Hospital Anxiety and Depression Scale
HbA1c: Glycosylated haemoglobin
HOMA: Homeostatic model assessment
ILI: Intensive lifestyle intervention
IRB: Institutional Review Board
ITT: Intention to treat
IWQoL-Lite: Impact of weight on quality of life questionnaire – lite
LED: Low-energy diet
NAFLD: Non-Alcoholic fatty liver disease
NYHA: New York Heart Association
OSA: Obstructive sleep apnoea
SAE: Serious adverse event
SD: Standard deviation
SIT-Q-7d: Sedentariness Questionnaire
T1DM: Type 1 diabetes mellitus
T2DM: Type 2 diabetes mellitus
UMC: Usual Medical Care
VLED: Very-low energy diet

## References

[1] Araghi MH, Chen YF, Jagielski A, Choudhury S, Banerjee D, Hussain S (2013). Effectiveness of lifestyle interventions on obstructive sleep apnea (OSA): systematic review and meta-analysis. Sleep.

[2] Grover SA, Kaouache M, Rempel P, Joseph L, Dawes M, Lau DCW, et al. Years of life lost and healthy life-years lost from diabetes and cardiovascular disease in overweight and obese people: a modelling study. Lancet Diab Endocrinol. 2015;3(2):114–22. 10.1016/S2213-8587(14)70229-3. 10.1016/S2213-8587(14)70229-3 25483220

[3] Young T, Peppard PE, Taheri S (2005). Excess weight and sleep-disordered breathing. J Appl Physiol.

[4] Jagielski AC, Brown A, Hosseini-Araghi M, Thomas GN, Taheri S (2014). The association between adiposity, mental well-being, and quality of life in extreme obesity. PLoS One.

[5] Dhanda N, Taheri S (2017). A narrative review of obesity and hearing loss. Int J Obes.

[6] Grant P, Piya M, McGowan B, Taheri S (2014). The bariatric physician. Clin Med (Lond).

[7] Ting SM, Nair H, Ching I, Taheri S, Dasgupta I (2009). Overweight, obesity and chronic kidney disease. Nephron Clin Pract.

[8] Pallayova M, Taheri S (2014). Non-alcoholic fatty liver disease in obese adults: clinical aspects and current management strategies. Clin Obes.

[9] Song SH, Hardisty CA (2009). Early onset type 2 diabetes mellitus: a harbinger for complications in later years--clinical observation from a secondary care cohort. QJM.

[10] Stevens J, Truesdale KP, Wang CH, Cai J, Erber E (2012). Body mass index at age 25 and all-cause mortality in whites and African Americans: the Atherosclerosis Risk in Communities study. J Adolesc Health.

[11] Strand BH, Kuh D, Shah I, Guralnik J, Hardy R (2012). Childhood, adolescent and early adult body mass index in relation to adult mortality: results from the British 1946 birth cohort. J Epidemiol Community Health.

[12] Tirosh A, Shai I, Afek A, Dubnov-Raz G, Ayalon N, Gordon B (2011). Adolescent BMI trajectory and risk of diabetes versus coronary disease. N Engl J Med.

[13] Schmidt M, Johannesdottir SA, Lemeshow S, Lash TL, Ulrichsen SP, Botker HE (2013). Obesity in young men, and individual and combined risks of type 2 diabetes, cardiovascular morbidity and death before 55 years of age: a Danish 33-year follow-up study. BMJ Open.

[14] Wilmot EG, Edwardson CL, Biddle SJ, Gorely T, Henson J, Khunti K (2013). Prevalence of diabetes and impaired glucose metabolism in younger ‘at risk’ UK adults: insights from the STAND programme of research. Diabet Med.

[15] Constantino MI, Molyneaux L, Limacher-Gisler F, Al-Saeed A, Luo C, Wu T (2013). Long-term complications and mortality in young-onset diabetes: type 2 diabetes is more hazardous and lethal than type 1 diabetes. Diabetes Care.

[16] Wong J, Constantino M, Yue DK (2015). Morbidity and mortality in young-onset type 2 diabetes in comparison to type 1 diabetes: where are we now?. Curr Diab Rep.

[17] Wong J, Molyneaux L, Constantino M, Twigg SM, Yue DK (2008). Timing is everything: age of onset influences long-term retinopathy risk in type 2 diabetes, independent of traditional risk factors. Diabetes Care.

[18] Gunathilake W, Song S, Sridharan S, Fernando DJ, Idris I (2010). Cardiovascular and metabolic risk profiles in young and old patients with type 2 diabetes. QJM.

[19] Henricsson M, Nystrom L, Blohme G, Ostman J, Kullberg C, Svensson M (2003). The incidence of retinopathy 10 years after diagnosis in young adult people with diabetes: results from the nationwide population-based Diabetes Incidence Study in Sweden (DISS). Diabetes Care.

[20] Choudhury S, Hussain S, Yao G, Hill J, Malik W, Taheri S (2013). The impact of a diabetes local enhanced service on quality outcome framework diabetes outcomes. PLoS One.

[21] Brown A, Guess N, Dornhorst A, Taheri S, Frost G (2017). Insulin-associated weight gain in obese type 2 diabetes mellitus patients: What can be done?. Diabetes Obes Metab.

[22] Zawiejska A, McAleese J, Yemparala P, Taheri S (2013). Treatment intensification in type 2 diabetes mellitus and obesity. Br J Gen Pract.

[23] Look ARG, Wing RR, Bolin P, Brancati FL, Bray GA, Clark JM (2013). Cardiovascular effects of intensive lifestyle intervention in type 2 diabetes. N Engl J Med.

[24] Wadden TA, Neiberg RH, Wing RR, Clark JM, Delahanty LM, Hill JO (2011). Four-year weight losses in the Look AHEAD study: factors associated with long-term success. Obesity.

[25] Wadden TA, West DS, Neiberg RH, Wing RR, Ryan DH, Johnson KC (2009). One-year weight losses in the Look AHEAD study: factors associated with success. Obesity.

[26] Look ARG, Pi-Sunyer X, Blackburn G, Brancati FL, Bray GA, Bright R (2007). Reduction in weight and cardiovascular disease risk factors in individuals with type 2 diabetes: one-year results of the look AHEAD trial. Diabetes Care.

[27] Look ARG, Wing RR (2010). Long-term effects of a lifestyle intervention on weight and cardiovascular risk factors in individuals with type 2 diabetes mellitus: four-year results of the Look AHEAD trial. Arch Intern Med.

[28] Buchwald H, Avidor Y, Braunwald E, Jensen MD, Pories W, Fahrbach K (2004). Bariatric surgery: a systematic review and meta-analysis. JAMA.

[29] Buchwald H, Estok R, Fahrbach K, Banel D, Jensen MD, Pories WJ (2009). Weight and type 2 diabetes after bariatric surgery: systematic review and meta-analysis. Am J Med.

[30] Schauer PR, Bhatt DL, Kirwan JP, Wolski K, Brethauer SA, Navaneethan SD (2014). Bariatric surgery versus intensive medical therapy for diabetes--3-year outcomes. N Engl J Med.

[31] Dixon JB, Hur KY, Lee WJ, Kim MJ, Chong K, Chen SC (2013). Gastric bypass in Type 2 diabetes with BMI < 30: weight and weight loss have a major influence on outcomes. Diabet Med.

[32] Dixon JB, O'Brien PE, Playfair J, Chapman L, Schachter LM, Skinner S (2008). Adjustable gastric banding and conventional therapy for type 2 diabetes: a randomized controlled trial. JAMA.

[33] Andrews RC, Cooper AR, Montgomery AA, Norcross AJ, Peters TJ, Sharp DJ (2011). Diet or diet plus physical activity versus usual care in patients with newly diagnosed type 2 diabetes: the Early ACTID randomised controlled trial. Lancet.

[34] Nanchahal K, Townsend J, Letley L, Haslam D, Wellings K, Haines A (2009). Weight-management interventions in primary care: a pilot randomised controlled trial. Br J Gen Pract.

[35] Wadden TA, West DS, Delahanty L, Jakicic J, Rejeski J, Look Ahead Research Group (2006). The Look AHEAD study: a description of the lifestyle intervention and the evidence supporting it. Obesity.

[36] Gregg EW, Chen H, Wagenknecht LE, Clark JM, Delahanty LM, Bantle J (2012). Association of an intensive lifestyle intervention with remission of type 2 diabetes. JAMA.

[37] Hu FB (2011). Diet and exercise for new-onset type 2 diabetes?. Lancet.

[38] Gloy VL, Briel M, Bhatt DL, Kashyap SR, Schauer PR, Mingrone G (2013). Bariatric surgery versus non-surgical treatment for obesity: a systematic review and meta-analysis of randomised controlled trials. BMJ.

[39] Sjostrom L, Narbro K, Sjostrom CD, Karason K, Larsson B, Wedel H (2007). Effects of bariatric surgery on mortality in Swedish obese subjects. N Engl J Med.

[40] Dixon JB, Chuang LM, Chong K, Chen SC, Lambert GW, Straznicky NE (2013). Predicting the glycemic response to gastric bypass surgery in patients with type 2 diabetes. Diabetes Care.

[41] Wang GF, Yan YX, Xu N, Yin D, Hui Y, Zhang JP, et al. Predictive Factors of Type 2 Diabetes Mellitus Remission Following Bariatric Surgery: a Meta-analysis. Obes Surg. 2015;25(2):199–208. 10.1007/s11695-014-1391-y. 10.1007/s11695-014-1391-y PMC4297287 25103403

[42] Anderson JW, Konz EC, Frederich RC, Wood CL (2001). Long-term weight-loss maintenance: a meta-analysis of US studies. Am J Clin Nutr.

[43] Astrup A, Rossner S (2000). Lessons from obesity management programmes: greater initial weight loss improves long-term maintenance. Obes Rev.

[44] Johansson K, Neovius M, Hemmingsson E (2014). Effects of anti-obesity drugs, diet, and exercise on weight-loss maintenance after a very-low-calorie diet or low-calorie diet: a systematic review and meta-analysis of randomized controlled trials. Am J Clin Nutr.

[45] Saris WH (2001). Very-low-calorie diets and sustained weight loss. Obes Res.

[46] Rolland C, Johnston KL, Lula S, Macdonald I, Broom J (2014). Long-term weight loss maintenance and management following a VLCD: a 3-year outcome. Int J Clin Pract.

[47] Perri MG (2014). Effects of behavioral treatment on long-term weight loss: lessons learned from the look AHEAD trial. Obesity.

[48] Purcell M, Sumithran P, Prendergast LA, Bouniu CJ, Delbridge E, Proietto J (2014). The effect of rate of weight loss on long-term weight management: a randomised controlled trial. Lancet Diab Endocrinol.

[49] Guidance N. Obesity Guidance CG189. National Institute for Nealth and Care Excellence; 2014. http://www.nice.org.uk/guidance/cg189. Accessed 12 May 2018.

[50] Tsai AG, Wadden TA (2006). The evolution of very-low-calorie diets: an update and meta-analysis. Obesity.

[51] Riecke BF, Christensen R, Christensen P, Leeds AR, Boesen M, Lohmander LS (2010). Comparing two low-energy diets for the treatment of knee osteoarthritis symptoms in obese patients: a pragmatic randomized clinical trial. Osteoarthritis Cartilage.

[52] Brown A, Frost G, Taheri S (2015). Is there a place for low-energy formula diets in weight management? British. J Obes.

[53] Brown A, Taheri S (2018). Very-low-energy diets for weight loss in patients with kidney disease. J Kidney Care.

[54] Bistrian BR, Blackburn GL, Flatt JP, Sizer J, Scrimshaw NS, Sherman M (1976). Nitrogen metabolism and insulin requirements in obese diabetic adults on a protein-sparing modified fast. Diabetes.

[55] Lean M, Brosnahan N, McLoone P, McCombie L, Higgs AB, Ross H (2013). Feasibility and indicative results from a 12-month low-energy liquid diet treatment and maintenance programme for severe obesity. Br J Gen Pract.

[56] Lim EL, Hollingsworth KG, Aribisala BS, Chen MJ, Mathers JC, Taylor R (2011). Reversal of type 2 diabetes: normalisation of beta cell function in association with decreased pancreas and liver triacylglycerol. Diabetologia.

[57] El Ouaamari A, Dirice E, Gedeon N, Hu J, Zhou JY, Shirakawa J (2016). SerpinB1 Promotes Pancreatic beta Cell Proliferation. Cell Metab.

[58] Pallayova M, Wilson V, John R, Taheri S (2013). Liver transplantation: a potential cure for hepatogenous diabetes?. Diabetes Care.

[59] Jakicic JM, Egan CM, Fabricatore AN, Gaussoin SA, Glasser SP, Hesson LA (2013). Four-year change in cardiorespiratory fitness and influence on glycemic control in adults with type 2 diabetes in a randomized trial: the Look AHEAD Trial. Diabetes Care.

[60] Curioni CC, Lourenco PM (2005). Long-term weight loss after diet and exercise: a systematic review. Int J Obes.

[61] Aguiar EJ, Morgan PJ, Collins CE, Plotnikoff RC, Callister R (2014). Efficacy of interventions that include diet, aerobic and resistance training components for type 2 diabetes prevention: a systematic review with meta-analysis. Int J Behav Nutr Phys Act.

[62] Umpierre D, Ribeiro PA, Kramer CK, Leitao CB, Zucatti AT, Azevedo MJ (2011). Physical activity advice only or structured exercise training and association with HbA1c levels in type 2 diabetes: a systematic review and meta-analysis. JAMA.

[63] Yang Z, Scott CA, Mao C, Tang J, Farmer AJ (2014). Resistance exercise versus aerobic exercise for type 2 diabetes: a systematic review and meta-analysis. Sports Med.

[64] Schwingshackl L, Missbach B, Dias S, Konig J, Hoffmann G (2014). Impact of different training modalities on glycaemic control and blood lipids in patients with type 2 diabetes: a systematic review and network meta-analysis. Diabetologia.

[65] Schwingshackl L, Dias S, Hoffmann G (2014). Impact of long-term lifestyle programmes on weight loss and cardiovascular risk factors in overweight/obese participants: a systematic review and network meta-analysis. Syst Rev.

[66] Balducci S, Sacchetti M, Haxhi J, Orlando G, D'Errico V, Fallucca S (2014). Physical exercise as therapy for type 2 diabetes mellitus. Diabetes Metab Res Rev.

[67] Association ACoSMatAD. Exercise and Type 2 Diabetes: American College of Sports Medicine and the American Diabetes Association: Joint Position Statement. Med Sci Sports Exerc. 2010;42(12):2282–303. 10.1249/MSS.0b013e3181eeb61c 21084931

[68] Committee CDACPGE (2014). Physical Activity and Diabetes.

[69] Pallayova M, Zaghloul HB, Arora T, Choudhury SM, Omar OM, Chagoury OL (2017). Investigating physiological glucose excursions before, during, and after Ramadan in adults without diabetes mellitus. Physiol Behav.

[70] Patel SR, Bellary S, Qin L, Gill PS, Taheri S, Heitmar R (2011). Abnormal retinal vascular function and lipid levels in a sample of healthy UK South Asians. Br J Ophthalmol.

[71] Wijndaele K, Godino JG, Lynch BM, Griffin SJ, Westgate K, I DEB (2014). Reliability and validity of a domain-specific last 7-d sedentary time questionnaire. Med Sci Sports Exerc.

[72] Arora T, Omar OM, Taheri S (2016). Assessment for the possibility of a first night effect for wrist actigraphy in adolescents. BMJ Open.

[73] Kolotkin RL, Crosby RD, Kosloski KD, Williams GR (2001). Development of a brief measure to assess quality of life in obesity. Obes Res.

[74] Araghi MH, Jagielski A, Neira I, Brown A, Higgs S, Thomas GN (2013). The complex associations among sleep quality, anxiety-depression, and quality of life in patients with extreme obesity. Sleep.

[75] Bliddal H, Leeds AR, Stigsgaard L, Astrup A, Christensen R (2011). Weight loss as treatment for knee osteoarthritis symptoms in obese patients: 1-year results from a randomised controlled trial. Ann Rheum Dis.

[76] Christensen P, Bartels EM, Riecke BF, Bliddal H, Leeds AR, Astrup A (2012). Improved nutritional status and bone health after diet-induced weight loss in sedentary osteoarthritis patients: a prospective cohort study. Eur J Clin Nutr.

[77] Pedersen LR, Olsen RH, Jurs A, Astrup A, Chabanova E, Simonsen L, et al. A randomised trial comparing weight loss with aerobic exercise in overweight individuals with coronary artery disease: The CUT-IT trial. Eur J Prev Cardiol. 2015;22(8):1009–17. 10.1177/2047487314545280. 10.1177/2047487314545280 25082954

[78] Jensen P, Zachariae C, Christensen R, Geiker NR, Schaadt BK, Stender S (2013). Effect of weight loss on the severity of psoriasis: a randomized clinical study. JAMA.

[79] Feinman RD, Pogozelski WK, Astrup A, Bernstein RK, Fine EJ, Westman EC, et al. Dietary carbohydrate restriction as the first approach in diabetes management: Critical review and evidence base. Nutrition. 2015;31(1):1–13. 10.1016/j.nut.2014.06.011. 10.1016/j.nut.2014.06.011 25287761

[80] Consultation WHOE (2004). Appropriate body-mass index for Asian populations and its implications for policy and intervention strategies. Lancet.

[81] Unick JL, Beavers D, Jakicic JM, Kitabchi AE, Knowler WC, Wadden TA (2011). Effectiveness of lifestyle interventions for individuals with severe obesity and type 2 diabetes: results from the Look AHEAD trial. Diabetes Care.

[82] LaRose JG, Leahey TM, Hill JO, Wing RR (2013). Differences in motivations and weight loss behaviors in young adults and older adults in the national weight control registry. Obesity.

[83] Lean ME, Leslie WS, Barnes AC, Brosnahan N, Thom G, McCombie L, et al. Primary care-led weight management for remission of type 2 diabetes (DiRECT): an open-label, cluster-randomised trial. Lancet. 2018;391(10120):541–51. 10.1016/S0140-6736(17)33102-1 29221645

[84] Tohid H, Choudhury SM, Agouba S, Aden A, Ahmed LHM, Omar O (2017). Perceptions and attitudes to clinical research participation in Qatar. Contemp Clin Trials Commun.

[85] Al-Kindi S, Al-Juhaishi T, Haddad F, Taheri S, Abi Khalil C (2015). Cardiovascular disease research activity in the Middle East: a bibliometric analysis. Ther Adv Cardiovasc Dis.

[86] Choudhury SM, Arora T, Alebbi S, Ahmed L, Aden A, Omar O (2016). How Do Qataris Source Health Information?. PLoS One.

[87] Butryn ML, Webb V, Wadden TA (2011). Behavioral treatment of obesity. Psychiatr Clin North Am.

